# Could dietary glutamate be contributing to the symptoms of obsessive-compulsive disorder?

**DOI:** 10.4155/fsoa-2017-0105

**Published:** 2018-01-10

**Authors:** Kathleen F Holton, Elizabeth W Cotter

**Affiliations:** 1 Department of Health Studies, American University, Washington DC 20016, USA; 2 Center for Behavioral Neuroscience, American University, Washington DC 20016, USA

**Keywords:** diet, glutamate, MSG, obsessive-compulsive disorder, OCD

## Abstract

A 50-year-old man who had suffered from daily obsessive-compulsive disorder (OCD) symptoms for 39 years, in addition to fibromyalgia and irritable bowel syndrome symptoms, was enrolled in a randomized double-blind placebo-controlled clinical trial to test the effects of a low-glutamate diet on fibromyalgia/irritable bowel syndrome symptoms. After 1 month on the low-glutamate diet all of his symptoms remitted, including his OCD, which had previously been nonresponsive to pharmacological treatment. This case study is limited by self-report of symptoms; however, glutamatergic neurotransmission appears to be dysregulated in OCD, suggesting biological plausibility for this observation. Future research is needed.

Obsessive-compulsive disorder (OCD) is a debilitating psychiatric condition characterized by uncontrollable obsessions (recurring obtrusive thoughts), resulting in anxiety and compulsions (repetitive behaviors used to alleviate anxiety caused by the obsessions) [1]. The prevalence of OCD has been estimated to be 1%, or approximately 3.1 million people in the USA [2]. OCD commonly co-occurs with other disorders such as fibromyalgia [3,4], which is a condition characterized by chronic widespread pain and other neurological symptoms such as fatigue, cognitive dysfunction and sleep problems [5]. Furthermore, one study estimated that approximately 35% of OCD patients met the criteria for irritable bowel syndrome (IBS), which is characterized by visceral pain accompanied by altered bowel habits [6]. All of these disorders share the potential for common etiology, as disordered glutamatergic neurotransmission has been implicated in all three conditions [7–10].

Due to altered glutamatergic neurotransmission being implicated in OCD, clinical trials are being conducted to test the effectiveness of pharmaceuticals which have the ability to modulate glutamate in the CNS [11]. Unfortunately, these medications (which are also used in fibromyalgia) tend to be limited in their usefulness due to significant side-effect profiles [12–14] and potential for abuse [15]. Alternative ways of normalizing glutamatergic neurotransmission are needed.

The objective of this paper is to describe the effect of a low-glutamate dietary intervention on one man's OCD symptoms, who was participating in a clinical trial examining the effects of dietary glutamate on fibromyalgia and IBS, and to discuss the potential implications for future research in OCD.

## Case report

A 50-year-old man was recruited for participation in a clinical trial examining the effect of dietary glutamate on fibromyalgia and IBS symptoms. All subjects were asked to report all symptoms that they were experiencing at baseline, then after 4 weeks on the dietary intervention, and at the end of each challenge week. In addition to his fibromyalgia and IBS symptoms, this man additionally reported OCD symptoms. He reported that he had experienced his OCD symptoms daily since he was 11 years old, and that they had always been nonresponsive to treatment. His obsessions revolved around the need to perfect certain movements, and his compulsions included the repetition of activities such as repeatedly getting up and down from a chair, in and out of the shower, etc. These compulsions took up a significant amount of time per day and interfered with his daily activities, including his ability to hold down a job.

The 50-year-old subject had undergone multiple unsuccessful pharmacological OCD treatments over the 39 years he had been experiencing symptoms. During his participation in the fibromyalgia study he was not currently taking medication for any of his symptoms (due to reportedly not tolerating the side effects of medications). Participation by individuals with a comorbid mental health diagnosis was deemed ethical by the research team and university IRB under certain circumstances. While individuals in acute mental health distress (e.g., active psychosis) were excluded from the study, participants with a mental health diagnosis that was stable for the past 6 months were eligible for participation.

**Figure 1.**
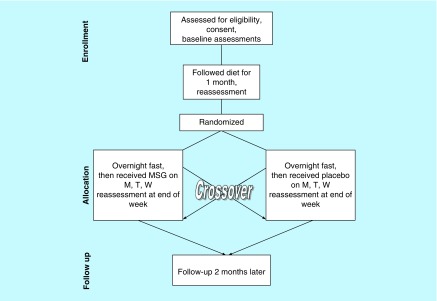
**Study design.** M: Monday; MSG: Monosodium glutamate; T: Tuesday; W: Wednesday.

Details about the study and overall results of this clinical trial in which he participated have been published previously [16]. However, a brief overview of the study design is included here (Figure 1).

After recruitment, all subjects received in-depth training on how to follow a 1-month low-glutamate diet, which restricted intake of common flavor enhancing food additives with free (i.e., not bound to a protein) glutamate. Foods with naturally occurring higher levels of free glutamate were also excluded from the diet (such as soy sauce and aged cheeses like parmesan). The additives excluded in this study are considered ‘excitotoxins’ due to their ability (when present in high enough amounts) to overexcite a neuron to the point that it dies [17,18]. Glutamate is the most ubiquitous neurotransmitter in mammalian systems, and disordered glutamatergic neurotransmission has been implicated in many disorders, including OCD [7,11,19–21], FM [8,9,16,22], and IBS [10,23–25]. The excitotoxin elimination diet used in this study required that subjects make one-for-one substitutions in the diet, replacing products which contained free glutamate, with other similar products which were free of these food additives, as opposed to improving the diet quality overall. For example, if a subject was consuming Doritos^®^, which can contain as many as 11 excitotoxins, they were asked to substitute these with simple corn tortilla chips, which have three ingredients: corn, oil and salt. This direct substitution removes the exposure to free glutamate, but does not increase the nutrient density of the diet.

After 1 month on the diet, subjects were again queried about their symptoms. Those who had >30% of their FM and IBS symptoms remit on the diet were eligible to go onto a double-blind, placebo-controlled, crossover challenge. Subjects came in fasting, and were randomized to receive monosodium glutamate (MSG) for three mornings of 1 week and placebo for 3 days of the other week. The main outcome measure was examining whether symptoms returned after each week of the crossover challenge. All fibromyalgia and IBS symptoms were formally assessed for remission (please see Holton *et al*. for a description of the measures [16]), while the subject's OCD symptoms were self-reported.

At the end of the 1-month excitotoxin elimination diet, the 50-year-old subject reported complete remission of all of his symptoms, including those related to his OCD. He expressed profound surprise at the remission of his OCD symptoms in particular, reporting that no medication had ever been able to help his symptoms during the past 39 years. During the 2 challenge weeks, his symptoms returned when challenged with MSG (week 1), and did not return when challenged with placebo (week 2). Over the 3 days of exposure during the MSG challenge week, all of his symptoms returned at the end of day 1 and peaked on the evening of day 3. The symptoms then slowly subsided over the following 4 days. He reported that the OCD symptoms experienced during the challenge week were typical, again presenting as needing to perfect movements (in addition to the return of his fibromyalgia and IBS symptoms). This response during the double-blind, placebo-controlled challenges confirmed that it was the removal of free glutamate from the diet, as opposed to other inadvertent dietary changes, that resulted in symptom improvement.

## Discussion

The above case study suggests the possibility that dietary free glutamate may be contributing to the symptoms of OCD, potentially through excessive excitation mediated by abnormal glutamatergic signaling. There is mounting evidence that OCD symptoms may be due to disordered glutamatergic neurotransmission. Below, we discuss what is currently known about glutamate and OCD, as well as the potential implications for future research in OCD.

### What is currently known

There is accumulating evidence implicating disordered glutamatergic neurotransmission in the etiology of OCD. Excellent reviews have been published recently which summarize abnormalities in glutamatergic neurotransmission in OCD from multiple perspectives [7,21]. Information is available summarizing the literature on genetic research examining glutamate-related genes [7,21,26], studies examining cerebrospinal fluid concentrations of glutamate and glycine (a co-agonist of the NMDA receptor) [7,21], magnetic resonance spectroscopy studies examining brain glutamate levels [7,20], as well as animal research [7,27] and clinical studies testing drugs which affect glutamatergic neurotransmission [11,28]. This emerging evidence over almost 2 decades of work suggests that dysregulation of glutamatergic neurotransmission may be playing a key role in the pathogenesis of OCD.

The use of medications which affect glutamatergic neurotransmission is currently being explored as a treatment option [11]. However, as mentioned earlier, these medications are limited by significant side effect profiles [12–14], and in the case of ketamine, also the potential for abuse [15]. Glutamate is the most ubiquitous neurotransmitter in the body, which makes pharmaceutical manipulation of glutamatergic neurotransmission very challenging. Thus, other ways of influencing glutamate in the CNS are needed. Dietary modulation of glutamate is a novel low-cost treatment method and has low risk of side effects. The case study presented herein suggests that future formal testing of a low-glutamate diet in OCD is warranted.

It is worth noting that the subject in this case report is part of the class of OCD patients who are nonresponsive to treatment. It has been estimated that 25–40% of OCD patients may fall into this group [10,29,30]. Noneffective treatment is frustrating for both patients and clinicians, thus finding other potential treatments is of great interest. Dietary change is attractive to patients since removing food additives from the diet does not confer risk of side effects. Thus, dietary change could be a cost effective and safe alternative, or adjunct treatment, in those with OCD if found to be efficacious in a larger trial. Future research is needed to test whether or not a low-glutamate diet can be used as an effective treatment for patients with OCD.

The observations reported in this case study can only be used for hypothesis generation. Since this man was recruited into a clinical trial studying fibromyalgia, no direct assessment of OCD symptoms was included in the measures collected. However, the double-blind placebo-controlled crossover challenge used in this study supports the idea that this man's OCD symptoms were actually responding to the low-glutamate diet. Thus, it is possible that other OCD patients may also respond to this diet. A future randomized controlled clinical trial testing a low-glutamate diet in OCD is needed to formally test this hypothesis.

## Future perspective

There is mounting evidence that disordered glutamatergic neurotransmission may be a key component of OCD symptomatology. To our knowledge, this case study is the first report of dietary exposure to free forms of glutamate being identified as a trigger for OCD symptoms. Formal clinical trials are needed to examine the potential impact of a low-glutamate diet in patients with OCD. In future research, we recommend including a ‘challenge’ phase, which includes the use of vegetarian capsules (as opposed to gelatin capsules, which are a source of glutamate) for testing whether challenge with MSG and placebo cause a return of symptoms after 1 month on the diet. If found to be effective in future randomized controlled trials, then this could cause an evolution in treatment protocols with the potential that the field could see dietary intervention being used as an adjunct treatment for OCD in the next 5–10 years.

**Executive summary**

- Obsessive-compulsive disorder (OCD) is observed to be a common comorbidity among those with fibromyalgia.
- Abnormal glutamatergic neurotransmission has been implicated in the etiology of OCD.
- This case study reports on a 50-year-old man who was participating in a clinical trial for fibromyalgia, who experienced complete remission of his OCD symptoms (in addition to his fibromyalgia symptoms) after 1 month on a low-glutamate diet, with a significant return of symptoms upon challenge with MSG, as compared with placebo, in a randomized controlled crossover clinical trial.
- This man's symptoms were reported to have been treatment resistant for the 39 years he had been experiencing OCD.
- A future randomized controlled clinical trial to formally test a low-glutamate dietary intervention in OCD is warranted.

## References

[1] Mcleod BD, Doss AJ, Ollendick TH (2013). *Diagnostic And Behavioral Assessment In Children And Adolescents: A Clinical Guide*.

[2] Kessler RC, Chiu WT, Demler O, Walters EE (2005). Prevalence, severity, and comorbidity of 12-month DSM-IV disorders in the National Comorbidity Survey Replication. *Arch. Gen. Psychiatry*.

[3] Uguz F, Çiçek E, Salli A (2010). Axis I and Axis II psychiatric disorders in patients with fibromyalgia. *Gen. Hosp. Psychiatry*.

[4] Raphael KG, Janal MN, Nayak S, Schwartz JE, Gallagher RM (2006). Psychiatric comorbidities in a community sample of women with fibromyalgia. *Pain*.

[5] Foa EB, Liebowitz MR, Kozak MJ (2005). Randomized, placebo-controlled trial of exposure and ritual prevention, clomipramine, and their combination in the treatment of obsessive-compulsive disorder. *Am. J. Psychiatry*.

[6] Moloney RD, Johnson AC, O'Mahony SM, Dinan TG, Greenwood-Van Meerveld B, Cryan JF (2016). Stress and the microbiota-gut-brain axis in visceral pain: relevance to irritable bowel syndrome. *CNS Neurosci. Ther.*.

[7] Pittenger C, Bloch MH, Williams K (2011). Glutamate abnormalities in obsessive compulsive disorder: neurobiology, pathophysiology, and treatment. *Pharmacol. Ther.*.

[8] Foerster BR, Nascimento TD, Deboer M (2015). Excitatory and inhibitory brain metabolites as targets of motor cortex transcranial direct current stimulation therapy and predictors of its efficacy in fibromyalgia. *Arthritis Rheumatol.*.

[9] Fayed N, Andres E, Viguera L, Modrego PJ, Garcia-Campayo J (2014). Higher glutamate+glutamine and reduction of N-acetylaspartate in posterior cingulate according to age range in patients with cognitive impairment and/or pain. *Acad. Radiol.*.

[10] Zhou Q, Price DD, Callam CS, Woodruff MA, Verne GN (2011). Effects of the *N*-methyl-D-aspartate receptor on temporal summation of second pain (wind-up) in irritable bowel syndrome. *J. Pain*.

[11] Pittenger C (2015). Glutamatergic agents for OCD and related disorders. *Curr. Treat. Options Psychiatry*.

[12] Jiang J, Jiang H (2015). Efficacy and adverse effects of memantine treatment for Alzheimer's disease from randomized controlled trials. *Neurol. Sci.*.

[13] Meador KJ, Baker GA (1997). Behavioral and cognitive effects of lamotrigine. *J. Child Neurol.*.

[14] Rasmussen KG (2014). Psychiatric side effects of ketamine in hospitalized medical patients administered subanesthetic doses for pain control. *Acta Neuropsychiatr.*.

[15] Bokor G, Anderson PD (2014). Ketamine: an update on its abuse. *J. Pharm. Pract.*.

[16] Holton KF, Taren DL, Thomson CA, Bennett RM, Jones KD (2012). The effect of dietary glutamate on fibromyalgia and irritable bowel symptoms. *Clin. Exp. Rheumatol.*.

[17] Olney JW, Labruyere J, De Gubareff T (1980). Brain damage in mice from voluntary ingestion of glutamate and aspartate. *Neurobehav. Toxicol.*.

[18] Olney JW, Zorumski C, Price MT, Labruyere J (1990). L-cysteine, a bicarbonate-sensitive endogenous excitotoxin. *Science*.

[19] Gasso P, Ortiz AE, Mas S (2015). Association between genetic variants related to glutamatergic, dopaminergic and neurodevelopment pathways and white matter microstructure in child and adolescent patients with obsessive-compulsive disorder. *J. Affect. Disord.*.

[20] Naaijen J, Lythgoe DJ, Amiri H, Buitelaar JK, Glennon JC (2015). Fronto-striatal glutamatergic compounds in compulsive and impulsive syndromes: a review of magnetic resonance spectroscopy studies. *Neurosci. Biobehav. Rev.*.

[21] Wu K, Hanna GL, Rosenberg DR, Arnold PD (2012). The role of glutamate signaling in the pathogenesis and treatment of obsessive-compulsive disorder. *Pharmacol. Biochem. Behav.*.

[22] Gerdle B, Larsson B, Forsberg F (2014). Chronic widespread pain: increased glutamate and lactate concentrations in the trapezius muscle and plasma. *Clin. J. Pain*.

[23] Moloney RD, O'Mahony SM, Dinan TG, Cryan JF (2015). Stress-induced visceral pain: toward animal models of irritable-bowel syndrome and associated comorbidities. *Front. Psychiatry*.

[24] Zhou L, Huang J, Gao J, Zhang G, Jiang J (2014). NMDA and AMPA receptors in the anterior cingulate cortex mediates visceral pain in visceral hypersensitivity rats. *Cell. Immunol.*.

[25] Mishra SP, Shukla SK, Pandey BL (2014). A preliminary evaluation of comparative effectiveness of riluzole in therapeutic regimen for irritable bowel syndrome. *Asian Pac. J. Trop. Biomed.*.

[26] Pauls DL, Abramovitch A, Rauch SL, Geller DA (2014). Obsessive-compulsive disorder: an integrative genetic and neurobiological perspective. *Nat. Rev. Neurosci.*.

[27] Grados M, Prazak M, Saif A, Halls A (2016). A review of animal models of obsessive-compulsive disorder: a focus on developmental, immune, endocrine and behavioral models. *Expert Opin. Drug Discov.*.

[28] Grados MA, Specht MW, Sung HM, Fortune D (2013). Glutamate drugs and pharmacogenetics of OCD: a pathway-based exploratory approach. *Expert Opin. Drug Discov.*.

[29] Bloch M, Landeros-Weisenberger A, Kelmendi B, Coric V, Bracken M, Leckman J (2006). A systematic review: antipsychotic augmentation with treatment refractory obsessive-compulsive disorder. *Mol. Psychiatry*.

[30] Pallanti S, Hollander E, Bienstock C (2002). Treatment non-response in OCD: methodological issues and operational definitions. *Int. J. Neuropsychopharmacol.*.

